# Quantification of intrapancreatic fat in type 2 diabetes by MRI

**DOI:** 10.1371/journal.pone.0174660

**Published:** 2017-04-03

**Authors:** Ahmad Al-Mrabeh, Kieren G. Hollingsworth, Sarah Steven, Dina Tiniakos, Roy Taylor

**Affiliations:** 1 Magnetic Resonance Centre, Institute of Cellular Medicine, Newcastle University, Newcastle upon Tyne, United Kingdom; 2 Department of Cellular Pathology, Royal Victoria Infirmary, Newcastle upon Tyne Hospitals and Institute of Cellular Medicine, Newcastle University, Newcastle upon Tyne, United Kingdom; Hirosaki Daigaku, JAPAN

## Abstract

**Objectives:**

Accumulation of intrapancreatic fat may be important in type 2 diabetes, but widely varying data have been reported. The standard quantification by MRI *in vivo* is time consuming and dependent upon a high level of experience. We aimed to develop a new method which would minimise inter-observer variation and to compare this against previously published datasets.

**Methods:**

A technique of ‘biopsying’ the image to minimise inclusion of non-parenchymal tissues was developed. Additionally, thresholding was applied to exclude both pancreatic ducts and intrusions of visceral fat, with pixels of fat values of <1% or >20% being excluded. The new MR image ‘biopsy’ (MR-opsy) was compared to the standard method by 6 independent observers with wide experience of image analysis but no experience of pancreas imaging. The effect of the new method was examined on datasets from two studies of weight loss in type 2 diabetes.

**Results:**

At low levels of intrapancreatic fat neither the result nor the inter-observer CV was changed by MR-opsy, thresholding or a combination of the methods. However, at higher levels the conventional method exhibited poor inter-observer agreement (coefficient of variation 26.9%) and the new combined method improved the CV to 4.3% (p<0.03). Using either MR-opsy alone or with thresholding, the new methods indicated a closer relationship between decrease in intrapancreatic fat and fall in blood glucose.

**Conclusion:**

The inter-observer variation for quantifying intrapancreatic fat was substantially improved by the new method when pancreas fat levels were moderately high. The method will improve comparability of pancreas fat measurement between research groups.

## Introduction

Type 2 diabetes never develops without substantial impairment of intrapancreatic insulin secretory capacity [1, 2]. This appears to be closely linked to increased pancreatic parenchymal fat with reduction of this depot during acute weight loss being specific to type 2 diabetes [3–7]. However, these studies are based on a time consuming, expert-dependent method of analysing magnetic resonance data, and other groups have reported a much wider range of values for pancreatic fat content in type 2 diabetes [8–16]. Controversy about results has followed [16, 17]. There is a pressing need for an easily reproducible method for precise measurement which will allow comparability between research centres. Adipose tissue expansion and intra organ fat accumulation are correlated with high levels of lipid inflammatory markers that cause oxidative stress in obesity and type 2 diabetes [18], and calorie restriction was reported to be associated with decrease in oxidative stress caused by lipid peroxidation [19]. The recent demonstration that exposure of pancreatic islets to increased fatty acids causes beta cell de-differentiation, and that this is the likely underlying mechanism for type 2 diabetes, further emphasizes the importance of precise quantification of pancreatic fat content [20, 21].

Magnetic resonance techniques allow non-invasive *in vivo* quantification of pancreatic fat [7, 13, 14, 22], other techniques having lower sensitivity [13, 23–26]. Values of greater than 20% have been reported [8, 9, 11], well in excess of histological estimation [24, 27]. Quantification of the percentage of fat within the parenchyma of the pancreas is challenging as it depends upon delineating tissue entirely within the organ. Our recent description of the involuted nature of the pancreas in type 2 diabetes offers some insight into the reported variability [28, 29]. The border of the normal pancreas is irregular, but far more so in type 2 diabetes. As the irregularity has been reported to be directly proportional to the fat content of the pancreas [28], it is possible that any inter-lobular intrusion of visceral fat might be interpreted as intra-pancreatic fat and hence overestimate the true value. Additionally, the volume of the pancreas is decreased by 30% in type 2 diabetes of recent onset and by 50% in type 2 diabetes of duration greater than 10 years [28, 29], making inclusion of visceral fat in any defined volume much more likely in type 2 diabetes compared with a non-diabetic group. This would obscure small differences in true parenchymal fat content and could explain at least part of the variability of intra-pancreatic fat content reported.

The original method of minimising inclusion of visceral fat intruding into the pancreas involved freehand drawing round a region to be sampled. This demands considerable experience and is extremely time consuming [5–7]. It is also prone to variation between observers. A simple rapid method which would yield consistent values between different observers would be of great benefit to further investigation of the role of intrapancreatic fat in the pathogenesis of type 2 diabetes. We have developed a new simplified method of analysing intrapancreatic fat content by MRI which minimises the extent of inclusion of extrinsic tissues. Additionally, we have applied the new method to previously published data sets [5, 6] to investigate whether its use would change the pathophysiological conclusions of previous work.

## Methods

### Pancreatic fat quantification

We collectively refer to intra-lobular and interlobular fat as “intrapancreatic fat” or “pancreas fat” as MRI cannot distinguish between these two fat compartments. This terminology is used throughout this paper.

MRI data were acquired using a 3.0 Tesla Philips Achieva scanner (Philips, Best, The Netherlands) with a 6 channel cardiac array for signal detection. The protocol consisted of matched breath-held acquisitions of (i) a 3 point Dixon acquisition to quantify the intrapancreatic triglyceride and (ii) a balanced turbo field echo image to aid anatomical delineation of the pancreas[29]. Another 3-point Dixon acquisition was prescribed at the level of the L4-L5 intervertebral space to estimate subcutaneous and visceral fat areas in this slice. The 3 point Dixon method [30]acquires three gradient-echo scans during one breath-hold with adjacent out-of-phase and in-phase echoes (repetition time/echo times/averages/flip angle = 50ms/3.45, 4.60, 5.75ms/1/5°, bandwidth 435Hz/pixel). Field-of-view was set according to patient size (400-480x300mm), zero filled to give a resolution of 1.39x1.40mm. 12 sections of 5mm thickness were used to image the pancreas during two 17-second breath-holds, while one section was acquired at L4-L5 Custom MATLAB software was used to model the fat and water contributions to the gradient echo signals using a spectral model of fat with 6 peaks based on [31] and a single R2* component. Proton density fat fraction maps (the fat signal expressed as a percentage of the total signal) were constructed taking account of noise bias[32]. The anatomical delineation was performed on a matched balanced turbo field echo (BTFE) image. BTFE images contain a mix of T_1_ and T_2_ contrast, which distinguishes high signal intensity from vessels with visceral fat with lower intensity signals from the pancreas. It can therefore be used to clearly delineate the boundaries of the pancreas from adjacent structures, including the surrounding visceral fat, the splenic vein, the superior mesenteric vessels the inferior vena cava and duodenum. Twelve axial sections of 5mm thickness were imaged during an eight second breath-hold (repetition time/echo time/flip angle = 3.1ms/1.6ms/40°, turbo factor 95, parallel imaging factor 2, bandwidth 1156Hz per pixel). The field of view and zero filled resolution were matched to the 3 point Dixon imaging.The conventional method of freehand drawing round an area to be within the substance of the pancreas and a newly developed MR image ‘biopsy’ method (MR-opsy) were compared. For both methods, the regions of interest were selected to be within the parenchymal tissues and avoiding areas of visceral fat, main blood vessels.

**Fig 1:**
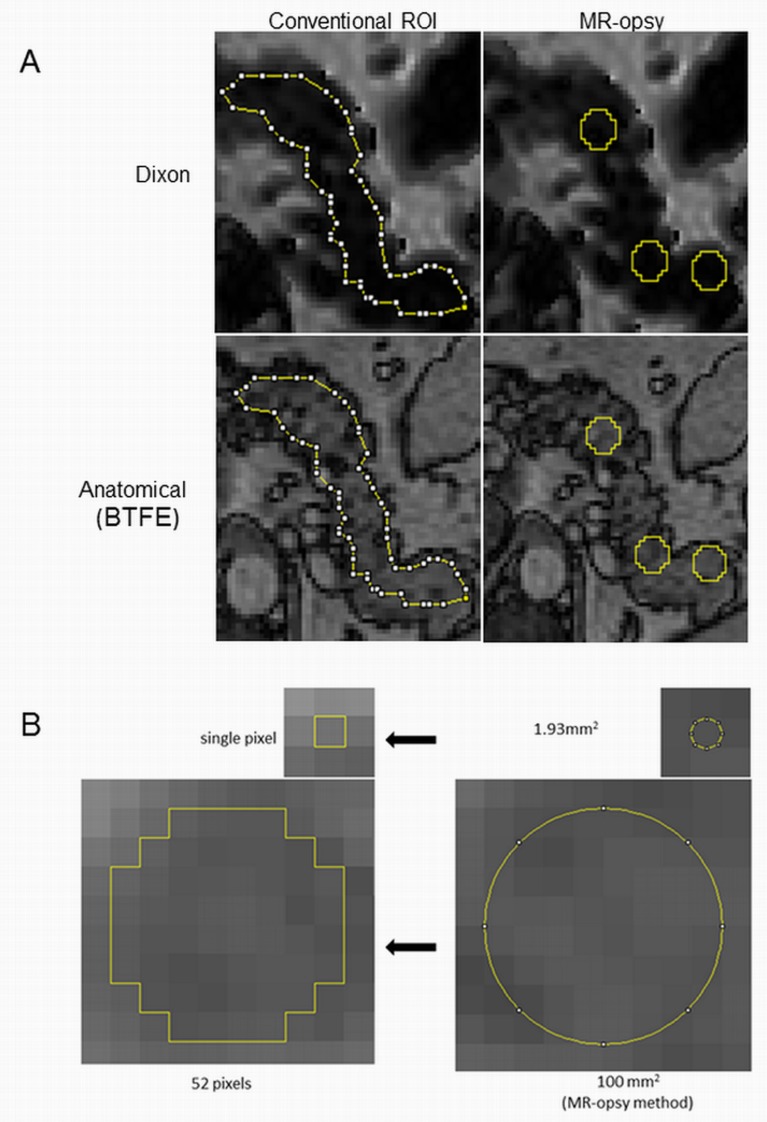
Illustration of sampling methods for intrapancreatic quantification. A: Representative MRI (3-point Dixon) slice of the pancreas was selected (upper panels). An anatomical scan was also acquired in parallel to the Dixon scan for localization purpose and for differentiation pancreas parenchymal tissue from main vessels and other adjacent abdomen tissues (lower panels). Regions of interest were carefully positioned away from pancreas borders to avoid contamination from visceral fat and away from main vessels. Conventional ROI: Polygon tool of ImageJ was used to select single ROI on the head, body and tail of pancreas away from visceral fat and main vessels. MR-opsy: Three ROIs (100mm^2^ each) were placed uniformly to represent different parts of the pancreas using ImageJ Oval tool away from visceral fat and main vessels. B: Magnified region of the pancreas to illustrate the size of biopsy selection (100mm^2^) relative to the size of an individual pixel (1.93mm^2^ = 1 pixel).The software reshape the oval selection (b, right) to take the nearest pixel shape (b, left).

For the conventional method, the ImageJ Polygon tool was used to select a region of interest in the parenchymal tissue of the pancreas head, body and tail. The region was selected to be as large as possible whilst being clear of the pancreas borders to avoid any possible contamination of surrounding visceral fat (Fig 1A).For MR-opsy, the Oval tool of ImageJ was used to select three regions of interest (~100 mm^2^ each) to represent equally the pancreas head, body and tail, the size of selection was chosen after pilot studies to permit easy placement entirely within the pancreas considering the irregularity in pancreas morphology (Fig 1A and 1B) [16]. In view of potential uneven distribution of parenchymal fat between different regions of the pancreas observed in some [33–37] but not all studies [12, 13, 38–40], sampling regions were placed equally throughout the pancreas to avoid possible bias. Analysis of both study datasets using the conventional methodology as originally published was carried out by experts experienced in pancreas anatomy. This was performed blinded to glucose tolerance and all clinical and metabolic markers both in the original studies and the present comparative study. Visceral and subcutaneous fat areas at L4-L5 were calculated from the L4-L5 proton density fat fraction map by thresholding and watershed analysis [41].

A step-by-step description of the process is presented in the Supplementary Methods section.

Two representative slices were selected to be assessed by each method and pancreatic fat content was calculated as the average pancreatic fat fraction of both slices.

### Thresholding of fat measurement

**Fig 2:**
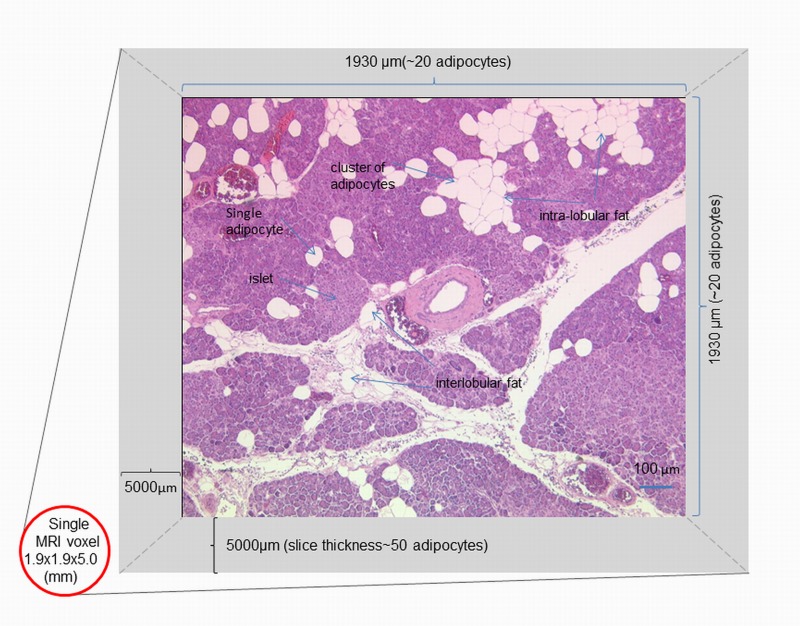
An illustration of adipocytes distribution within single MRI voxel of parenchymal tissues of pancreas. Histological section of background normal pancreatic tissue of a 48-year female undergoing pancreatectomy for a neuroendocrine tumour. The average size of single adipocyte is approximately 100μm, but adipocytes can occur in clusters. Based on average adipocyte size of 100μm, the maximum number of adipocytes likely to be present in one voxel is ~400x50 = 20000 adipocytes. The upper threshold of 20% assumes that the maximum number to be 4000 adipocytes within a single voxel of pancreas.

Each image slice through the pancreas is 5mm thick to permit an adequate signal to noise ratio in the fat fraction images. In order to eliminate potential contribution of non- parenchymal tissue (visceral fat, pancreatic duct or blood vessel) within the selected region, a threshold was applied to both methods by collecting the histogram data within the area of selection and computing the resulted data to exclude pixels values outside the threshold limits which would otherwise contribute to the mean value (see step-by-step description in S1 Methods). Anonymised histological sections of pancreatic parenchymal tissues from people undergoing pancreatic surgery taken from various locations in the pancreas showed adipocyte distribution similar to the upper limit of 20% reported by Pinnick et al [27]. Hence, the maximum number of adipocytes clustered within a single voxel of pancreatic parenchymal tissues is estimated to be approximately 4000 (Fig 2), and any MRI fat signal above 20% is likely to be due to contamination by visceral fat tissue.

**Fig 3:**
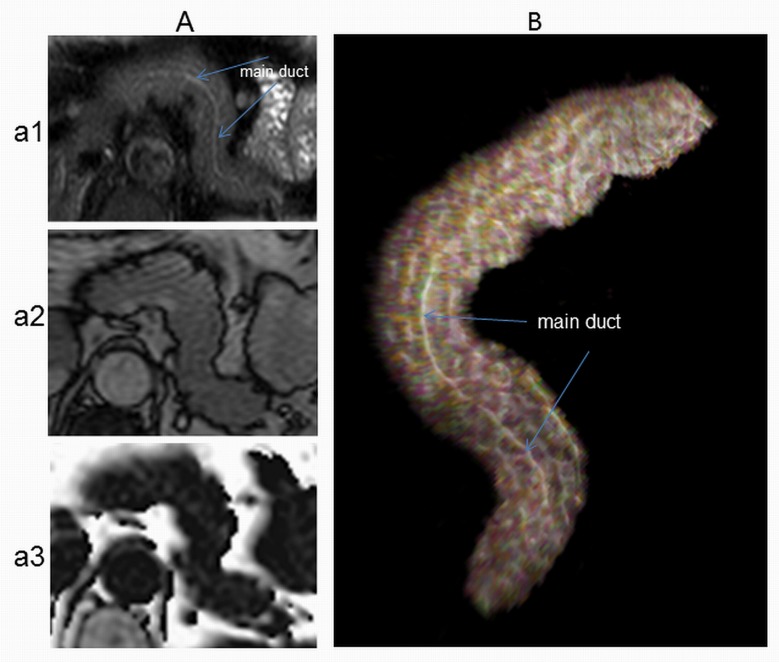
Example of ductal system architecture within the pancreas. A: different MRI axial acquisitions of the pancreas in T2DM subject (a1: T2-SPAIR, a2: BTFE, a3: 3-point Dixon). Pancreas of T2-SPAIR (a1) sequence was segmented and volume rendered in Drishti as described before [28], volume rendered image was colour tagged then opacity level was manipulated to show the distribution of pancreatic ductal system in white colour (Drishti version 2.6.3).

**Fig 4:**
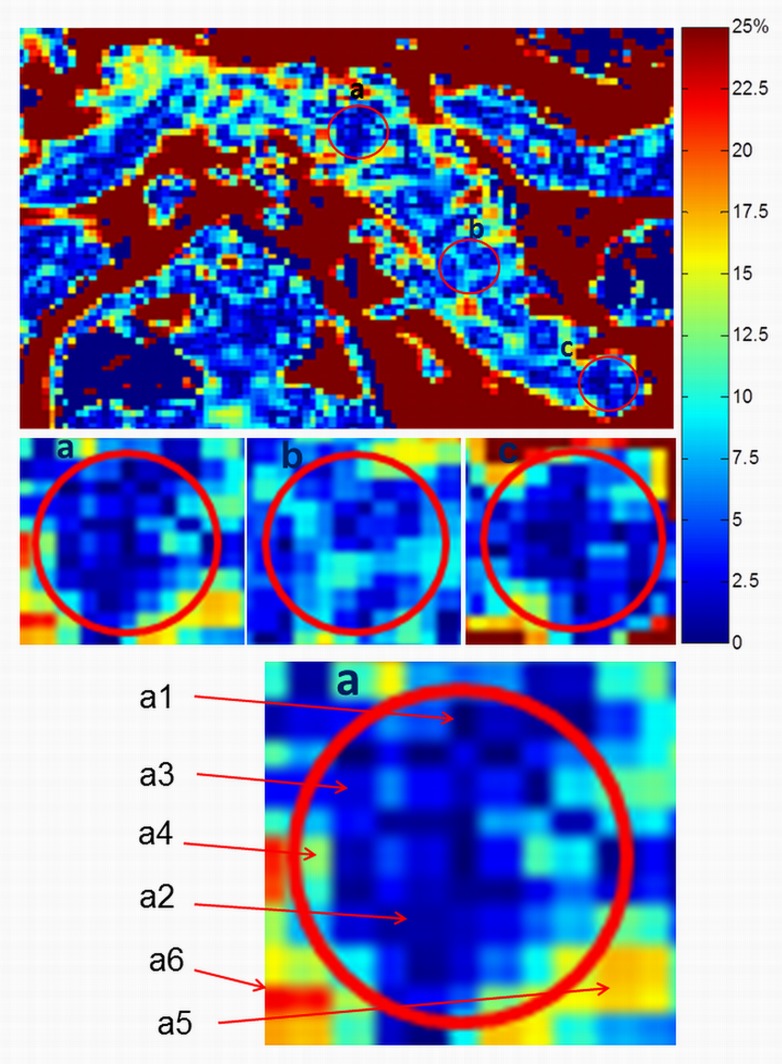
Colour map of pancreatic fat distribution in type 2 diabetes. The colour map shows the wide range of fat distribution within the sampling area. This underlies the rationale for thresholding to exclude non-parenchymal tissues. The colour bar on the right shows fat level from 0% (dark blue) to 25% (red). Threshold levels were set to exclude areas of fat content less than 1% (possible blood vessels or main duct) or above 20% (visceral fat contamination). Parenchymal fat was considered to range between 1–20%. a1-a6 represent areas of varied fat content within the single MR-opsy selection (a1: < 1%, a2: 1–5%, a3:6–10%, a4:11–15%, a5:16–20%, a6: > 20%).

Similarly, pixels almost devoid of fat (<1%) are likely to represent major pancreatic ducts or blood vessels, and these cannot be discriminated on the BTFE image. The main pancreatic duct network is suggested though not segmentable on T2-weighted images, such as Fig 3A1 (a T2 weighted fast spin echo, TR/TE = 946ms/70ms with spectrally selective adiabatic inversion recovery, SPAIR, for fat saturation). A thresholding range of 1–20% was therefore applied to the original data, and the performance of both methods was compared before and after thresholding. The rationale is explained in Fig 4.

### Reproducibility of fat quantification

Type 2 diabetes participants with low (3.3%) and high (6.5%) pancreas fat levels were analysed to test the reproducibility of methods. MR scans acquired prior to weight loss were examined by 6 independent observers using the methods in random order. The observers have wide experience of image analysis but no previous experience of assessing pancreas. Each was asked to follow instructions and quantify intrapancreatic fat by both methods. Coefficient of variation (CV) was calculated for the 6 independent measurements and compared by both methods for the two participants.

### Intervention studies design

The Counterbalance study tested the durability of type 2 diabetes reversal after a very low calorie diet in a group of 30 people with 0.5–23 years of diabetes duration [5]. Intrapancreatic fat was quantified at baseline, after 8 weeks of very low calorie diet (VLCD) and following a 6 months weight maintenance programme. Participants were considered as responders if fasting plasma glucose level <7mmol/l following VLCD and return to normal diet [5].

The bariatric surgery study evaluated change in intrapancreatic fat after weight loss at 8 weeks post-surgery in groups with (n = 18) or without (n = 9) type 2 diabetes [6].

### Statistical analysis

Minitab 17 (Minitab Inc, State College, PA, USA) was used for statistical analysis. All data are presented as mean ±SEM, and *p* value <0.05 was considered statistically significant. Student’s paired *t*-test was used to measure significance.

## Results

### Inter-observer agreement

**Fig 5:**
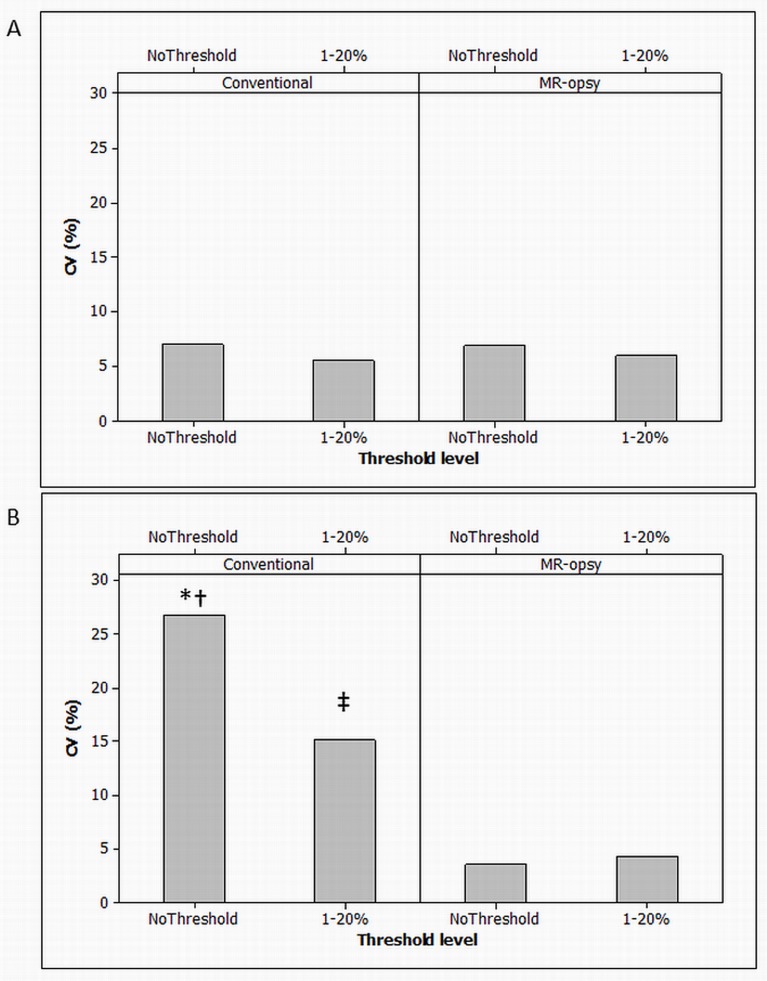
Reproducibility of fat quantification methods. The inter-observer variation for each method is shown for low level (3%) of pancreatic fat (A), and high level (6%) of pancreatic fat (B). Data for both methods are shown with and without 1–20% thresholding. * p<0.05 Conventional vs MR-opsy before thresholding. ‡ p<0.05 Conventional vs MR-opsy after thresholding. † p<0.05 Conventional without thresholding vs with thresholding.

**Fig 6:**
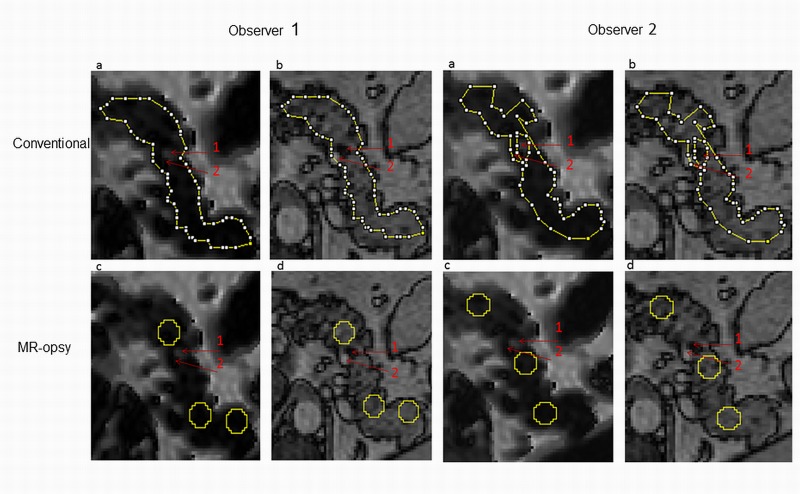
Example of areas selected by two observers using conventional and MR-opsy methods. ROIs of participants with the high level of pancreatic fat (6%) were shown using conventional (a,b), and MR-opsy (c,d) methods for observers 1 and 2. Two regions with potential contribution to wrong estimation of pancreatic fat content were selected: Region 1 represents a region of focal fat which appears bright on the Dixon scan (a,c), and dark on the anatomical BTFE scan (b,d). Region 2 represents blood vessel and appears dark on the Dixon scan (a,c), and bright on the anatomical scan (b,d). It is clear that observer 1 included both areas within the selection using conventional method whereas biopsy method avoided those regions by the same observer.

At low levels of intrapancreatic fat, neither the result nor the inter-observer CV (coefficient of variation) was changed by MR-opsy, thresholding or a combination of the methods (Fig 5A). At high levels of pancreatic fat, the conventional method used by non-expert observers exhibited poor inter-observer precision (CV 26.9%; Fig 5B). Application of the MR-opsy method improved the CV to 4.3% (p<0.03; Fig 5B). The components of the improvement were separately assessed. MR-opsy alone improved the precision (CV 3.5%; *p* = 0.02) as did application of thresholding but to a lesser extent (CV 15.2%; *p*<0.05). Areas of selections by the observers were investigated in order to understand the difference in CV between the methods despite similarity in mean intrapancreatic fat percentage. It was found that some observers were more or less conservative in their perception of the boundary of the pancreas and the visceral fat or areas of blood vessels that could lead to both over-estimation or under-estimation of pancreatic fat content, respectively (Fig 6).

### Effect of fat quantification method on counterbalance study data

At baseline, using the conventional method in expert hands, there was no significant difference in intrapancreatic fat between those who subsequently were or were not able to reverse their T2DM by weight loss (5.3±0.4% vs. 5.9±0.7%; Table 1). Application of MR-opsy, with or without thresholding did not change this (Table 1).

**Table 1:** Counterbalance study: Pancreas fat change in responders and non-responders before and after weight loss.

Method & body characteristics	responders (n = 12)			non-responders (n = 17)		
	baseline	8 weeks	6 months	baseline	8 weeks	6 months
**Conventional ROI**	5.3±0.4	4.5±0.3*	4.4±0.3*	5.9±0.7	5.3±0.6*	5.0±0.5*
**MR-opsy method**	4.5±0.3	4.0±0.3*	3.7±0.3*	5.5±0.8	5.5±0.6	4.9±0.6
**Conventional (1–20%)**	5.7±0.4	5.0±0.2*	4.9±0.3*	6.0±0.4	5.7±0.4	5.5±0.4*
**MR-opsy (1–20%)**	5.1±0.3	4.5±0.3*	4.4±0.3*	5.6 ±0.5	5.7±0.5	5.3±0.5
**Body weight (kg)**	99.8±3.2	84.1±3.1 *	84.4±3.2*	96.7±3.9	83.6±3.5*	84.8±3.7*
**BMI (kg/m**^**2**^**)**	34.0±0.8	28.6±0.8 *	28.7±0.7*	34.4±1.1	29.8±1.1*	30.2±1.1*
**Visceral fat (cm**^**2**^**)**	287.0 ±23.1	191.9 ±18.9*	238.6 ± 20.3*	289.6 ±23.7	209.5 ± 22.1*	198.9 ± 4.8*
**Subcutaneous fat (cm**^**2**^**)**	319.6 ± 31.0	232.0 ± 23.1*	238.6 ± 20.3*	285.4 ± 24.7	223.3 ± 23.5*	219.3 ± 22.8*

Data are presented as mean ± SEM

*p<0.05 vs baseline.

Responders: fasting plasma glucose <7mmol/l, non-responders: fasting plasma glucose >7mmol/l.

After the 8 week weight loss period using the conventional method there was a significant fall in intrapancreatic fat in both responder and non-responder groups. Use of MR-opsy, with or without thresholding did not change the significance of the decrease in intrapancreatic fat in the responders. In the non-responders the significant fall reported by the conventional method was not observed by any of the new methods (Table 1).

After the 6 month weight maintenance period, using the conventional method there was a significant fall in intrapancreatic fat in both responder and non-responder groups. In the responders, all methods observed the previously reported significant decrease in intrapancreatic fat content. In the non-responders, using MR-opsy with or without thresholding no significant change was observed (Table 1). Use of thresholding alone in the non-responders decreased the apparent extent of fall in pancreas fat (Table 1).

### Effect of fat quantification method on bariatric surgery study

At baseline, use of any of the methods showed intrapancreatic fat to be significantly higher in the group with type 2 diabetes compared with the normal glucose tolerant group (conventional: 6.6±0.5% vs. 5.1±0.2%; MR-opsy with thresholding: 6.4±0.3 vs. 5.1±0.6%; Table 2). The fall in intrapancreatic fat during weight loss in the type 2 diabetic group remained significant using all methods. Conversely, weight loss brought about no change within the NGT group between baseline and 8 weeks after surgery using conventional or new methods (Table 2).

**Table 2:** Bariatric surgery study: Pancreas fat change in type 2 diabetes (T2DM) and normal glucose tolerance (NGT) participants before and after weight loss.

Method & body	T2DM (n = 16)		NGT (N = 8)	
characteristics	baseline	8 weeks	baseline	8 weeks
**Conventional ROI**	6.6±0.5†	5.4±0.4*	5.1±0.2	5.5±0.4
**MR-opsy method**	6.0±0.4†	5.5±0.4*	4.6±0.7	5.3±0.5
**Conventional (1–20%)**	6.9±0.4†	6.0±0.3*	5.5±0.2	6.0±0.4
**MR-opsy (1–20%)**	6.4±0.3†	5.8±0.3*	5.1±0.6	5.5±0.4
**Body weight (kg)**	121.1±3.0	104.5±2.7*	114.5±5.0	99.7±4.6*
**BMI (kg/m**^**2**^**)**	42.7±0.7	36.9±0.7*	41.3±1.0	36.4±0.8*
**Visceral fat (cm**^**2**^**)**	300.4±17.5	241.3±11.0*	244.5±28.4	187.9±28.3*
**Subcutaneous fat (cm**^**2**^**)**	453.8±28.9	393.2±26.8*	496.4±16.0	409.7±26.0*

Data are presented as mean ± SEM

*p<0.05 vs baseline

†p<0.05 T2DM versus NGT

### Variability of fat distribution within the pancreas areas

Fat distribution varied significantly between the head and other parts of the pancreas for the Counterbalance study (Table 3). At the baseline of the study, fat percentage was higher in the head of the pancreas compared with the body or the tail using conventional, MR-opsy alone or MR-opsy with thresholding methods indicating heterogeneity among different pancreatic tissues in fat distribution (MR-opsy plus thresholding: head vs. body, p = 0.006; head vs. tail, p = 0.01).

**Table 3:** Fat% in different regions of the pancreas before and after intervention studies.

Pancreas region	Study	baseline		2 months		6 months	
		MR-opsy	plus 20%	MR-opsy	plus 20%	MR-opsy	plus 20%
Head	Counterbalance (n = 29)	5.5±0.4*†	5.7±0.3*†	5.1±0.4*†	5.5±0.3*†	4.6±0.4*	5.0±0.3*†
Body		4.9±0.5	5.1±0.3	4.7±0.4	5.1±0.3	4.3±0.4	4.7±0.3
Tail		4.9±0.5	5.2±0.3	4.7±0.5	5.0±0.4	4.2±0.4	4.7±0.3
Mean		5.2±0.5	5.4±0.3	4.9±0.4	5.2±0.3	4.4±0.4	4.8±0.3
Head	Bariatric surgery (n = 16)	6.0±0.4	6.5±0.4	5.4±0.5	5.3±0.5	-	-
Body		6.0±0.4	6.5±0.4	5.9±0.5	5.6±0.5	-	-
Tail		6.0±0.6	6.3±0.5	5.1±0.4	5.3±0.5	-	-
Mean		6.0±0.4	6.4±0.4	5.5±0.4	5.7±0.3	-	-
Head	Control (n = 8)	4.9±0.8	5.4±0.7	5.8±0.6*	5.9±0.5	-	-
Body		4.6±0.9	5.1±0.8	5.6±0.6†	5.6±0.5	-	-
Tail		4.3±0.5	4.6±0.3	4.4±0.5	4.9±0.4	-	-
Mean		4.6±0.6	5.1±0.6	5.3±0.5	5.5±0.4	-	-

The minor difference compared with some reported means in the manuscript is due to the mean being taken from calculating or thresholding the three ROIs, together whereas each ROI was processed separately in the tabulated data.

Data ± SEM

*p<0.05 vs body

† p<0.05 vs tail.

### Correlation between pancreas fat and some body characteristics

No correlation between body weight and BMI with pancreatic fat was found in the Counterbalance study using any quantification method. In the bariatric surgery study, there was correlation between pancreatic fat and weight using the conventional method only(r = 0. 5, p = 0.04). There was no correlation observed between fat content and age in both studies. Interestingly, we found significant correlation between pancreatic fat and diabetes duration within the Counterbalance study (r = 0.48, p = 0.008).

## Discussion

Reproducible quantification of intra-parenchymal pancreas fat is important to allow comparisons between data from different research groups, and this is especially important as absolute differences in pancreas fat between type 2 diabetes and normal are modest [5–7]. We demonstrate that higher inter-observer agreement can be achieved using MR-opsy compared with the conventional region of interest method when intrapancreatic fat levels are higher and pancreas volume is lower [28]. As intrapancreatic fat increases and pancreas volume decreases with increasing disease duration [28, 29], the data are of particular relevance to this disease state. Re-analysis using the new method of previously published intervention studies of type 2 diabetes, which used conventional methodology applied by experts, did not change the previously reported pathophysiological implications.

Several studies have demonstrated the association between increased intrapancreatic fat and type 2 diabetes. In diabetes-prone rodent models of type 2 diabetes overfeeding brings about impairment of beta cell function, and this susceptibility to lipid availability is reflected in studies on isolated islets [3, 4, 42–44]. In humans predisposed to develop type 2 diabetes, prolonged Intralipid infusion severely impairs beta-cell function [45]. Conversely, removal of excess lipid from the environment of the pancreatic islet allows return of normal insulin secretion in early type 2 diabetes [5, 7]. This has also been observed in isolated islets [3]. The apparent relationship of this lipid depot to the pathophysiology of type 2 diabetes emphasizes the importance of methodology for precise measurement.

Homogeneity of fat distribution within the pancreas is a topic of great debate [12, 13, 33–40]. The series of studies on people with type 2 diabetes showed a degree of variability in fat content between head, body and tail. Given that the biological relevance of this work is to investigate any effect of fat upon overall beta cell function and that these are distributed throughout the pancreas, inclusion of data from each region in a mean to represent the whole pancreas is justified in order to represent fat distribution in the whole pancreas. Although selection of one region could be sufficient under certain conditions of homogeneous pancreas fat distribution such as in the study of very obese people, use of the MR-opsy method is still appropriate. A potential disadvantage of the method could arise if there was marked heterogeneity of fat content between regions of the pancreas, but the present observations and those of others suggest that this is rare. The pancreas in type 2 diabetes is 30–50% smaller than normal [28, 29]. The decrease in volume as diabetes duration increases is accompanied by notable increase in irregularity in the pancreas borders. This implies greater likelihood of inclusion of the extra-pancreatic fat which exists between lobules [28]. The contribution of pancreatic ducts or blood vessels which cannot be identified in the image, has previously been overlooked. It is notable that the mean level of pancreas fat increased as a result of 1–20% thresholding (Tables 1 and 2). The conventional method of pancreas fat quantification using magnetic resonance imaging has resulted in a wide range of reported pancreas fat content [7, 13, 46] and the present data suggest that this would be minimised by use of MR-opsy. In the present study the observers, who were experienced in image analysis but not in studying the pancreas, reported that placement of the 100 mm^2^ MR-opsies was not challenging, and was also rapid (approximately 5 minutes vs. up to 30 minutes for conventional drawing round a region of interest).

Short duration type 2 diabetes can be reversed after weight loss with restoration of normal beta cell function and this has been reported to be associated with a fall in intrapancreatic fat content [5–7]. Application of the new method resulted in identification of no change in intrapancreatic fat in the longer duration group (which did not respond to weight loss by normalising plasma glucose). These subjects had smaller, more irregular pancreases than the responders, and the new method is more likely to reflect true intra-pancreatic fat levels. In the bariatric study, the type 2 diabetes participants exhibited a good return to normal glucose control [6], and quantitation of intrapancreatic fat by either convention method in expert hands or by the new method showed a significant decrease.

Several studies reported the robustness of MR-based fat quantification methods [22, 47, 48]. A recent phantom study evaluated the reproducibility of MRI fat quantification technique between research centres, MR scanner vendors, field strengths, and acquisition protocols [49] emphasizing the importance of standardized image analysis technique for precise comparison. However, use of a phantom does not reflect the complexities introduced by variable inclusion of visceral fat and fluid filled intra-organ ducts.

The published studies employing MR to quantify pancreas fat content used a wide range of methods for sampling size and location selected for fat quantification (Table 4). This can partially explain the discrepancy in reported pancreas fat content. Of these studies, some reported a significant relationship between diabetes or insulin resistance and pancreas fat [6, 8–12, 14, 15, 29, 40, 50, 51]. Other studies reported no significant difference in pancreas fat content between type 2 diabetes and non-diabetic controls [7, 10, 13, 37, 52, 53].

**Table 4:** Summary of up-to-date studies employed MR for fat quantification in the pancreas.

Reference	Method	Participants	Sample size	Sample region	Fat content		Significance (T2DM vs. control)
					T2DM	Control	
Kovanlikaya et al. (2005)	MRI	15 NGT(14–17 years- 6 lean/9 obese)	ROI = 3x not specified size	tail	N/A	total: 30.1±14.6% lean: 15.6±2.6% obese:39.7±10.4%	N/A
Tushuizen et al. (2007)	MRS	12 T2DM/24 NGT	VOI = (1.0x1.0x2.0)cm	body/tail	20.4% (13.4–43.6%)	9.7% (7.0–20.2)	p<0.05
Kim et al. (2007)	MRI	retrospective analysis of 135 patients	visual inspection	head	N/A	5 with focal fatty replacement	N/A
Schwenzer et al. (2008)	MRI	17 at risk of T2DM (BMI 31.7 kg/m^2^)	ROI = 3x (1.0–1.5 cm^2^)	head/body /tail	N/A	8.8% ± 5.7%	N/A
Lingvay et al. (2009)	MRS	11 T2DM/23 IGT/45 NGT	VOI = (10x10x20)mm	body	T2DM:5.5% IGT:5.6%	BMI<25: 0.5% BMI ≥25: 3.2%	p<0.05
Hu et al. (2010)	MRI	8 NGT	VOI = (10x10x8)mm-(10x20x12) mm	largest possible position-matching MRS/MRI	N/A	MRI~5.0%	N/A
	MRS					MRS~8.0%	
Heni et al. (2010)	MRI	28 NGT/23 IGT	ROI = 3x (1.0–1.5 cm^2^)	head/body/tail	IGT:8.3±3.5%	7.4±2.3%	p>0.05
Sijens et al. (2010)	MRI	36 NGT (8 obese) (BMI 27.5 kg/m^2^)	ROI = (1x 2.68 cm^2^)	tail	N/A	normal: 2.3% obese: 3.6%	N/A
van der Zijl et al. (2011)	MRS	16 NGT/29 IFG/19 IFG-IGT	VOI = (2.5x1.0x1.0)cm	body/tail	IFG:12.1% (5.1–17.5%), IGT: 22.4% (7.3–36.2%)	7.6% (2.9–13.4%)	p<0.05
Lim et al. (2011)	MRI	11 T2DM/9 NGT (VLCD intervention)	ROI = varied size	head/body/tail	8.0±1.6%	6.0±1.3%	p>0.05
Li et al. (2011)	MRI	126 healthy men (BMI≤ 25 kg/m^2^) A = 20–50 years, B = 50–70 years	ROI = 3x(0.4–0.6 cm^2^)	head/body/tail	N/A	A = 2.8± 0.7%, B = 6.3 ± 1.2%	N/A
Le et al. (2011)	MRI	138 obese (74 Hispanics/64 Africans)	all pancreas slices	head/body/tail	N/A	Hispanics:7.3±3.8% /Africans: 6.2±2.6%	N/A
Szczepaniak et al. (2012)	MRS	100 overweight (20 Black,50 Hispanic, 30 White)	VOI = (10x10x20)mm	body/tail	N/A	Black~2.2% Whites~5.6% Hispanics~5.8%	N/A
Targher et al. (2012)	MRI	42 obese/ BMI 35.2 kg/m^2^	ROI = 3x circles (1–2 cm diameter or less)	head/body/tail	N/A	11% (7–22%)	N/A
Patel et al. (2013a)	MRI	43 NAFLD: (15 T2DM/28 without diabetes)	ROI = 1-2x 100 mm^2^	head/body/tail	7.9%	8.8%	p>0.05
Patel et al. (2013b)	MRI	43 NAFLD/49 healthy	1–2 ROIs (100 mm^2^)	head/body/tail	NAFLD:8.5% IR:7.3%	healthy:3.6% NIT:4.5%	p <0.05
Livingstone et al. (2014)	MRS	24 healthy: 52.6± 18 years /BMI = 25.8kg/m^2^	VOI = (2.0 x1.0x1.0)cm	body	N/A	5.5 ± 5.9%	N/A
	MRI		ROI-1 = (34 x 32 x 34) mm, ROI-2 = (102 x 96x 102) mm	head/body/tail	N/A	ROI-1 = 11.1%, ROI-2 = 8.0%	N/A
Ma et al. (2014)	MRI	24 T2DM/10 healthy	ROI = (10x10x10) mm	head	15.4±12.2%	4.9±1.3%	p<0.05
	MRS		VOI = (10x10x10) mm		18.2±12.5%	6.9±1.6%	p<0.05
Wong et al. (2014)	MRS	685 NAFLD screening study/33 with T2DM	N/A	body	21/33≤10.4% 12/33>10.4%	5.5% (3.8–8.7%) 90% (1.8–10.4%)	N/A
Cohen et al. (2014)	MRI	50 healthy children /(8–18 years/BMI 29kg/m^2^)	ROI = (12x12x12) mm	tail	N/A	1.5%±3.44 (0–14%)	N/A
Gaborti et al. (2015)	MRS	19 T2DM/13 lean/13 obese	VOI = (17x15x15)mm	body	23.8±3.2%	obese14.0±3.3%	p<0.05
						lean 7.5±0.9%	p<0.05
Macauley et al. (2015)	MRI	41 T2DM/14 NGT	ROI = varied size	head/body/tail	5.4 ± 0.3%	4.4 ± 0.4%	p<0.05
Wicklow et al. (2015)	MRS	20 Youth-onset T2DM/34NGT	VOI = (3.0x3.0x3.0)cm	tail	2.4%	1.2%	p>0.05
Pacifico et al.(2015)	MRI	158 obese children /(18 pre-diabetes/80 with NAFLD)	ROI = 1-2x(1.0 cm^2^)	head/body/tail	3.6% (1.7–5.5%)	1.9% (1.3–3.1%)	p<0.05
Begovatz et al. (2015)	MRS	14 T2DM/14 IGT/28 NGT	VOI = (20x10x10)mm (total fat)	body/tail	8.4% [5.6, 13.1%]	1.95% [0.3, 6.4%]	p<0.05
	MRI		ROI = 2x(100mm^2^) (parenchymal fat)	head/body/tail	0.4% [-0.3, 0.7%]	0.14% [-0.1, 0.4%]	p>0.05
Kühn et al. (2015)	MRI	740 NGT/430 IGT/70 T2DM	ROI = 3x(varied size)	head/body/tail	4.6% [2.8, 6.4%]/ IGT:4.5% [3.9, 5.1%]	4.4% [4.1,4.8%]	p>0.05
Idilman et al. (2015)	MRI	41 NAFLD (5 with T2DM)	ROI = 3x(1.0x1.0x1.0) cm	head/body/tail	12.2±12%	4.8±3.5%	p<0.05
Chai et al. (2016)	MRI	70 T2DM/30 NGT	ROI = 158.46/ 154.37/ 156.47 mm^2^	head/body/tail	5.2±3.8%	3.5±2.0%	p<0.05
Steven et al. (2016a)	MRI	29 T2DM (VLCD intervention)	ROI = varied size	head/body/tail	5.7± 0.5%	N/A	N/A
Steven et al. (2016b)	MRI	18 T2DM/ 9 NGT (bariatric surgery)	ROI = varied size	head/body/tail	6.6±0.5%	5.1±0.2%	p<0.05

MRS: Magnetic Resonance Spectroscopy, MRI: Magnetic Resonance Imaging, T2DM: Type 2 Diabetes Mellitus, NAFLD: Non-alcoholic fatty liver disease, ROI: region of interest, VOI: volume of interest, NGT: normal glucose tolerance, IGT: impaired glucose tolerance, IFG: impaired fasting glucose, IR: insulin resistance, NIT: normal insulin tolerance. Mean± SD / Mean±SEM were presented in most studies; median and quartile were used for skewed data. Difference was considered statistically different at the level of 0.05%; different statistics were used to derive the *p* values. Different scanners and fat/water separation methods were applied. For both MRS/MRI studies, careful positioning of the VOI/ROI away from the vessels and visceral fat was reported. In MRS studies, visceral fat contaminated spectra were excluded and the mean percentage of several spectra per VOI was used. In MRI: different sampling approaches were followed by selecting ROIs, and majority of studies presented the mean percentage of more than one ROI.

The remaining studies did not compare between non-diabetic and diabetic groups [6, 22, 35, 38, 39, 46, 54–60]. Whereas magnetic resonance imaging methods allow subsequent selection of the volume to analyse, magnetic resonance spectroscopy depends upon acquiring data from a volume of the body pre-selected by imaging. Consequently it is particularly susceptible to inclusion of visceral fat due to respiratory and other movement in the scanner. Such spectroscopy methods tend to report higher pancreas fat content (up to 24%). Hu *et al*. [22] reported that MR spectroscopy was less accurate than imaging for pancreas fat quantification due to the difficulty in voxel positioning, and this is consistent with the present data on the effect of selection of region of interest for fat quantification. By combining (a) selection of several regions to represent tissues from the whole organ; (b) restriction of size of the selected region to decrease contamination from visceral fat; and (c) applying thresholding to exclude contribution from non-parenchymal tissues, an improvement in inter-observer agreement is observed.

The major limitation of the current study is the lack of a gold standard for non-invasive quantitation of fat solely within the parenchymal tissue of the pancreas. At present, neither Dixon nor anatomical scans can differentiate between parenchymal tissue and ductal or small vascular structures in the pancreas. Optimization of image acquisition for differentiating between those small structures is demanding and currently being developed. For example the T2- SPAIR sequence used in image 3 might allow exclusion of the main pancreatic duct within the MR-opsy selection. As one of the major limiting factors for resolution of pancreas imaging is breath-hold duration, development of sparse scanning techniques which acquire data more rapidly may be expected to permit higher resolution imaging [61]. Under condition of severe pancreas fat infiltration of parenchymal tissues, the performance of the MR-opsy method alone can be limited. Nonetheless, the proposed 20% threshold to exclude areas of visceral fat invasion remains useful under such circumstances, and values close to 20% should trigger detailed examination of the pancreas anatomy when selecting regions of interest. In conclusion, quantification of fat within the pancreas by MRI is significantly affected by the method of sampling and the new MR-opsy method allows higher inter-observer agreement. Application of this standardised new method with thresholding should permit measurement of changes in true intrapancreatic fat content which can reliably be compared between different research groups.

## Supporting information

S1 Methods (DOC)

## Abbreviations

VLCD: Very Low Calorie Diet
MRI: Magnetic Resonance Imaging
ROI: Region of Interest
T2DM: Type 2 Diabetes Mellitus
