# Supplementary material for: Quantification of intrapancreatic fat in type 2 diabetes by MRI
Source: PLoS One. 2017 Apr 3;12(4):e0174660. doi: 10.1371/journal.pone.0174660 (PMC5378354; doi:10.1371/journal.pone.0174660)
Supplement: S1 Methods — (DOC) [file pone.0174660.s001.doc]

**Standard Operating Procedure for** **quantification of intrapancreatic fat using MRI**

# Background

Accumulation of fat within the pancreas is central for type 2 diabetes development, but varying data have been reported from different research groups using MRI. The standard assessment by segmenting the whole pancreatic cross-section area is time consuming and dependent upon a high level of experience. A new method was developed and by thresholding, pixels with fat values of <1% or >20% were excluded in order to minimise inclusion of non-parenchymal tissues. The new MR image ‘biopsy’ (MR-opsy) is simple and reproducible for better comparison between research centres.

# Materials

Processed fat fraction image of the pancreas derived from 3-point Dixon sequence [1] or equivalent which we refer to as the “fat fraction image”. The pixels contain values between 0 to 100%.

A co-located, registered Balanced Turbo Field Echo (BTFE) image highlighting the anatomy of the pancreas which we refer to as the [1] “anatomical image”. The detailed acquisition parameters for both sequences are contained in reference [1].

The freely available ImageJ software from the NIH [2].

Excel spreadsheet template for automated thresholding of Histogram data.

**The right arrow (→) is used throughout the SOP to indicate mouse selections (“left click”) when navigating through ImageJ commands.**

# Procedures

- 1. **Placement of the MR-opsy**.
- Use ImageJ to open the “fat fraction image” and the “anatomical image” simultaneously (better to have two screens attached to the PC for easy navigation through the two image stacks).
- Identify the two candidate slices which meet the criteria for selection including: being centrally located within the pancreas in the foot-head direction and having sufficient area to place the 100mm^2^ ROI distributed over head, body and tail of the pancreas.
- Using ImageJ Oval tool, design a circular region of~100mm^2^ (MR-opsy), and label it as ROI-01within the ROI Manager window (ImageJ→ Analyze →Tools→ ROI Manager), then copy it twice and label as ROI-02 and ROI-03.

**Highlight the name of the ROI**, **and using the mouse move the selected ROI freely to compare the area of selection on both the fat fraction image and anatomical image. Once you are happy with your selection click the Update button within the ROI Manager main panel.**

- Repeat the same for the second slice then save all the ROIs (the “RoiSet” file in ImageJ terminology) under a specific name in the desired folder.

**Important: The above process is ONLY required once, then you can copy the RoiSets file across to another desired folder but modify the file name, selected slices and ROI positions.**

Highlight the three ROI selections on the first slice at once (ctrl+shift+ mouse click) then select from the ROI Manager main panel: More →OR (Combine) → Add (this will combine the three ROIs in one file, see illustrative below). The software will create a random name to the new file, highlight the name → Rename → label the new file that contains the three combined ROIs as “average-slice-01”.


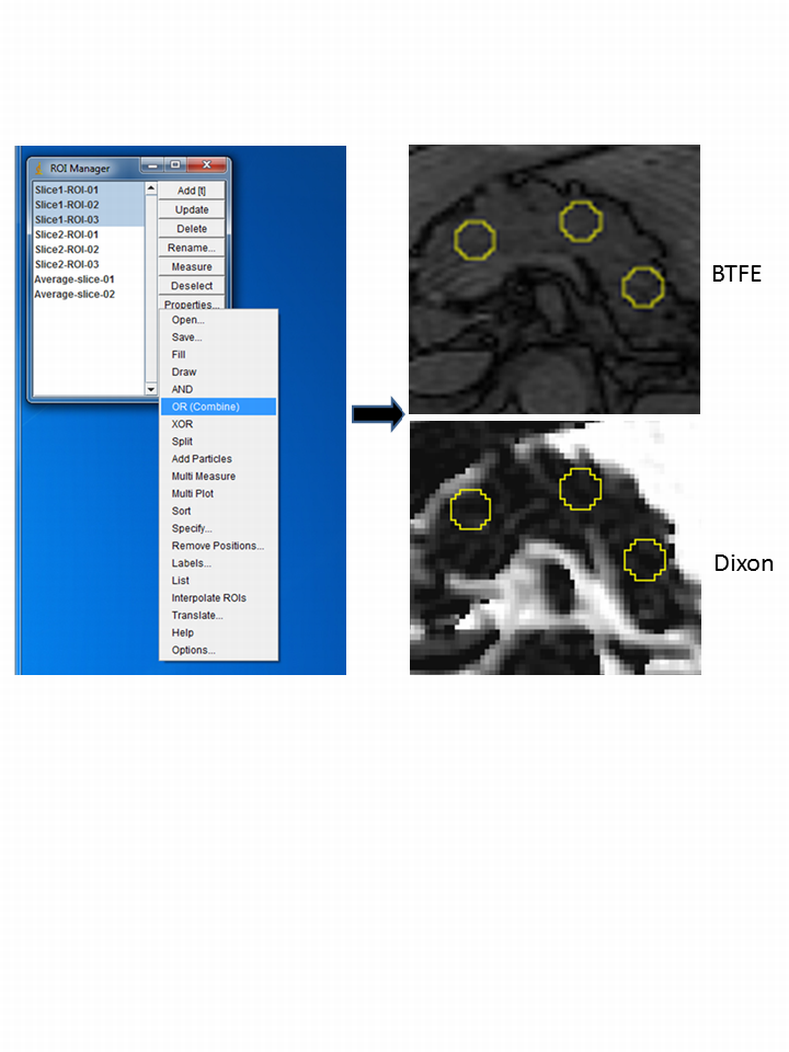


**Step 1: MR-opsy selection**

- Repeat the same for the second slice, and label as “average-slice-02”.

Highlight all the RoiSets in the ROI Manager and update the saved RoiSet file (More →Save →select the file name and overwrite it).

**You need first to go to ImageJ**→ **Analyze** → **Set Measurements, and then enable settings including display label, area of selection, maximum/ minimum gray value, and mean gray value.**

- From ImageJ main window click Analyze → Measure to get the percentage fat for all highlighted RoiSets within the ROI Manager (make sure that you click on the fat fraction image not anatomical map before you do this step).
- Save the result as spreadsheet within the desired folder by selecting File→ Save As (this will generate pancreatic fat without excluding any values).

**3.2 Thresholding of the selection**

- Highlight average-slice-01 from the ROI manager window, and select Analyze →Histogram→OK, a new window of the Histogram data of selected area will appear (see illustrative example below).
- From the Histogram window click List →File → Save As (the software will save it as a spreadsheet under the name: Histogram of the original file name.
- Use Excel spreadsheet to exclude values less than 1% and over 20% from final fat

percentage (see illustrative example below).


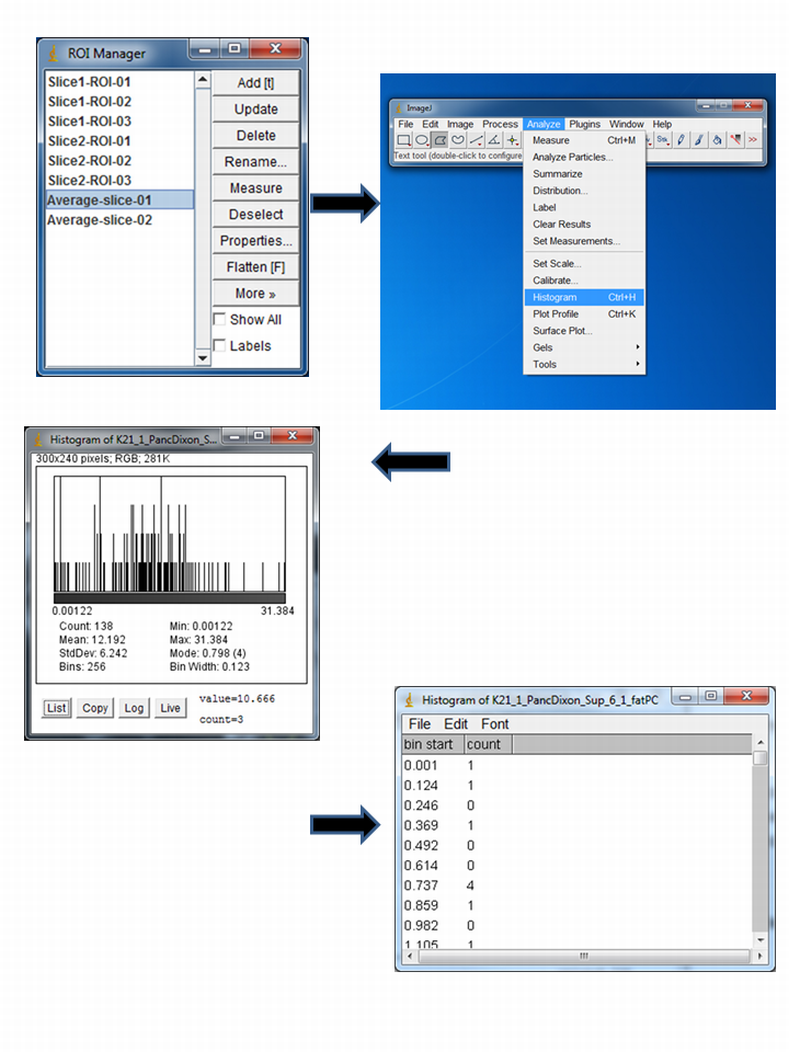


**Step 2: Thresholding (Histogram data)**


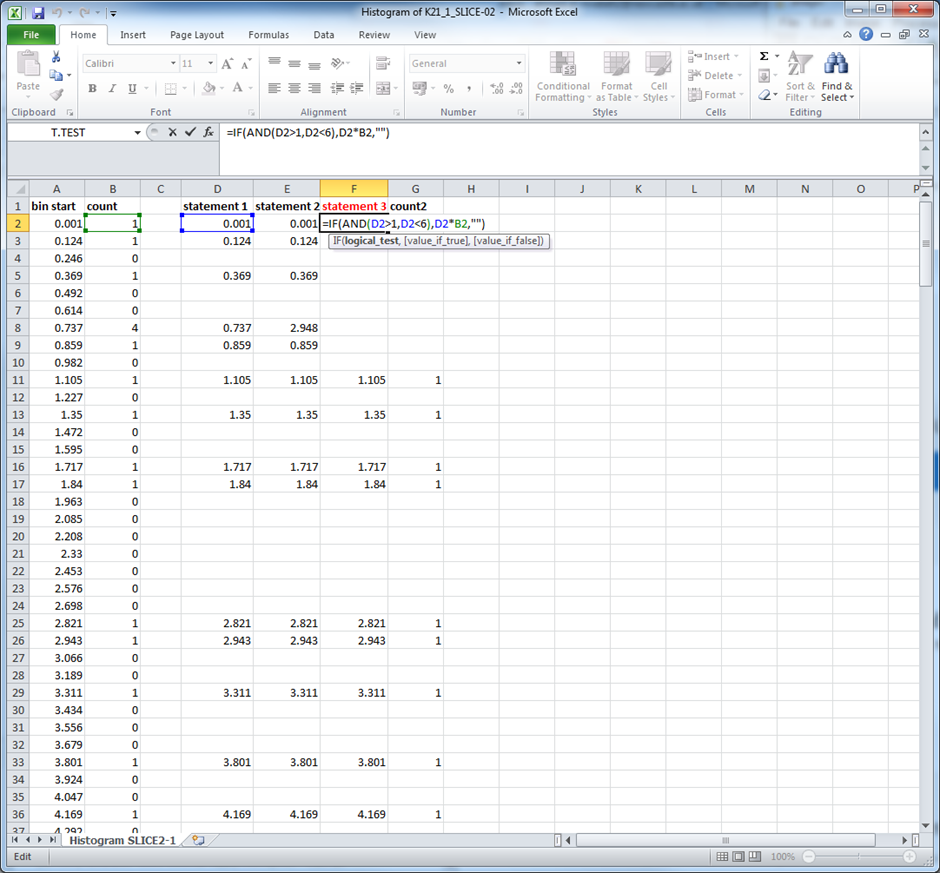


**Step 3: Excluding pixels outside thresholding limit**

**Important: you need to do this spreadsheet ONLY once and use it as a template then from the histogram window copy the data across to the empty template (from Histogram window click List→Edit→Select All→Copy→ paste in the template spreadsheet).**

**4. Outline flowchart of the procedures**


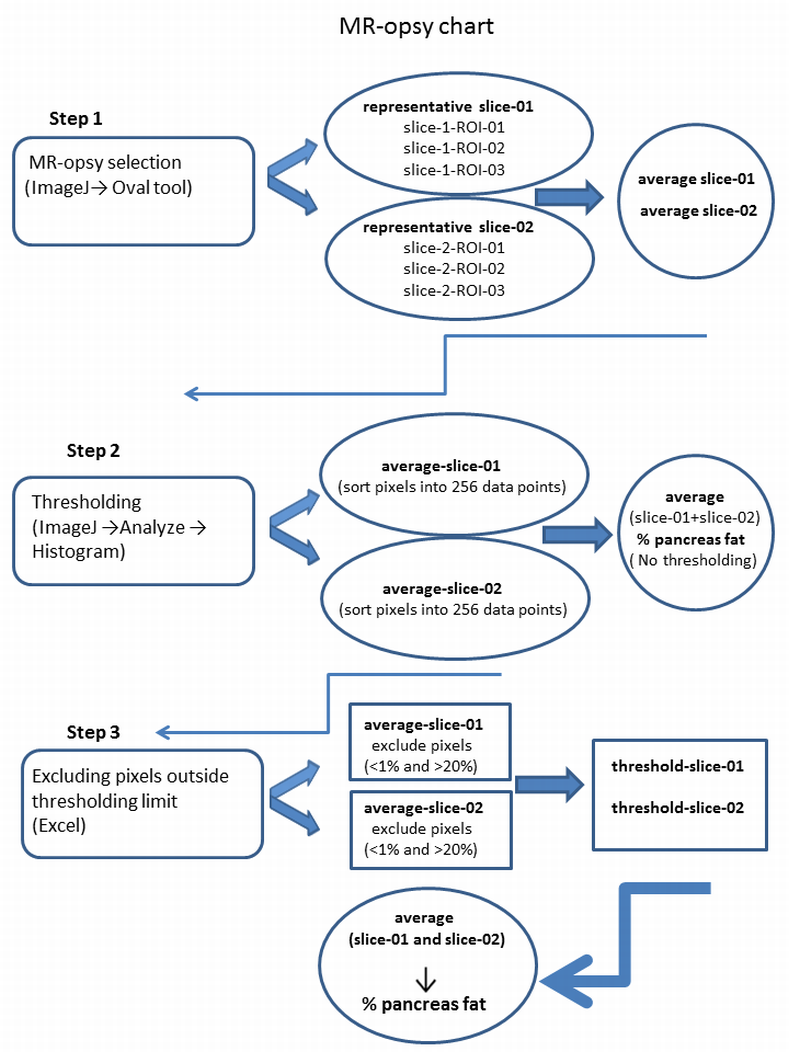


**5. References**

[1] Macauley M, Percival K, Thelwall PE, Hollingsworth KG, Taylor R (2015) Altered volume, morphology and composition of the pancreas in type 2 diabetes. PloS one 10: e0126825

[2] Schneider CA, Rasband WS, Eliceiri KW (2012) NIH Image to ImageJ: 25 years of image analysis. Nature methods 9: 671-675
